# Prevalence, molecular characterization, and histopathological impact of *Trichomonas gallinae* in domestic pigeons from Northeastern Egypt

**DOI:** 10.1038/s41598-025-12854-2

**Published:** 2025-08-04

**Authors:** Al-Shaimaa M. Sadek, Doaa S. Farghaly, Tasneme A. Ghazy

**Affiliations:** 1 Parasitology, Zoology and Entomology Department, Faculty of Science, Al-Azhar University (Girl’s Branch), Nasr City, Cairo, Egypt; 2 Medical Entomology, Zoology and Entomology Department, Faculty of Science, Al-Azhar University (Girl’s Branch), Cairo, Egypt; 3 M.Sc., Zoology and Entomology Department, Faculty of Science, Al-Azhar University (Girl’s branch), Cairo, Egypt

**Keywords:** DNA sequencing, Egypt, Epidemiology, Histopathology, PCR, Phylogenetic analysis, *Trichomonas galliinae*, Genetics, Zoology

## Abstract

*Trichomonas gallinae* (*T. gallinae*) is a single-celled flagellate protozoan that causes trichomoniasis, a serious and widespread infectious disease primarily affecting Columbiformes. This study investigated the prevalence, molecular characterization, and histopathological effects of *T. gallinae* in domestic pigeons (*Columba livia domestica*) from different environments and regions in Egypt. A total of 685 pigeons were examined from markets, dovecotes, and houses across Cairo, Giza, and Qalubyya Governorates from February 2022 to November 2024. Microscopic examination confirmed an infection in 533 pigeons, yielding an overall infection rate of 77.8%. Markets exhibited the highest prevalence (91.8%), followed by dovecotes (72.1%) and houses (58.4%). Regionally, Cairo recorded the highest infection rate (80.7%), followed by Giza (76.0%) and Qalubyya (76.0%). Seasonal variation indicated that summer accounted for the highest number of cases (48.2%), while winter had the lowest (11.4%). Age distribution revealed a strong predominance of squabs (70.7%) compared to adults (29.3%), and gender analysis showed a significantly higher prevalence in females (83.4%) than in males (16.6%). Histopathological examination of oropharyngeal mucosa, proventriculus, and gizzard showed distinct pathological changes, including severe necrosis, caseation, and granulomatous tissue reactions, which are pathognomonic for *T. gallinae* infection. Molecular analysis confirmed the presence of *T. gallinae*, with PCR amplification of the ITS1/5.8S/ITS2 gene revealing two novel strains, were deposited in GenBank with accession numbers (OR498119) and (OR498120). These strains exhibited high nucleotide sequence identity with isolates from China, Germany, and Spain, indicating a high degree of genetic conservation and a widespread global distribution of *T. gallinae*. These findings highlight the widespread prevalence of *T. gallinae* in domestic pigeons, particularly in urban and commercial environments, with seasonal, age, and gender-related variations influencing infection rates. The detection of novel genetic variants and severe tissue damage emphasizes the need for enhanced surveillance, control measures, and further research on the pathogenicity and epidemiology of *T. gallinae* in pigeons and potential spillovers to other avian hosts.

**Supplementary Information:**

The online version contains supplementary material available at 10.1038/s41598-025-12854-2.

## Introduction

Pigeons and doves are widely distributed across the world, and their relationship with humans’ dates back centuries^1^. Domestic pigeons (*Columba livia domestica*) have been utilized for food, kept as pets, and revered as cultural and religious symbols for centuries^2^. Due to their close interaction with humans and other bird species, pigeons can serve as reservoirs of infectious pathogens, including *T. gallinae*, a protozoan parasite responsible for avian trichomoniasis^3^. *Trichomonas gallinae* is a unicellular protozoan that colonizes the upper digestive tract of clinically and sub clinically infected pigeons. This parasite causes canker, a disease that primarily affects the crop, esophagus, liver, and lungs^4^. Transmission occurs through contaminated food, water, and direct contact, particularly via regurgitated crop secretions during feeding^5^. Given its potential zoonotic risk and the role of pigeons in urban and rural ecosystems, ongoing surveillance of *T. gallinae* is essential for disease control and management^6^. The prevalence of *T. gallinae* infection varies significantly across different regions, depending on climatic conditions, host susceptibility, and environmental factors^7^. Despite its widespread occurrence, the epidemiology of *T. gallinae* in Egypt remains poorly characterized, highlighting the need for further research on risk factors, geographic distribution, and molecular diversity of circulating strains. In Egypt, several studies have examined the occurrence of *T. gallinae* in pigeons and other birds, often using morphological or microscopic methods for diagnosis^8,9^. However, molecular data remain limited, especially regarding the diversity and phylogenetic relationships of circulating strains in specific regions such as northeastern Egypt. Additionally, the histopathological manifestations of *T. gallinae* infection in naturally infected birds in this part of the country are not well described, despite their significance for understanding disease progression and severity.

Histopathological examination provides critical insights into tissue-level damage and host immune responses associated with *T. gallinae* infections. The parasite induces necrosis, ulceration, and granulomatous lesions, primarily in the crop, esophagus, and proventriculus. In severe cases, the infection may disseminate to the liver and other organs, leading to systemic complications^8^. Histopathological findings, such as inflammatory cell infiltration, epithelial degeneration, and submucosal thickening, offer a deeper understanding of host–pathogen interactions and disease progression^10,11^.

Advances in molecular diagnostics have improved the detection and classification of *T. gallinae* strains. PCR-based assays targeting the internal transcribed spacer (ITS) region, including the ITS1-5.8S-ITS2 rRNA gene have been widely used to analyze the genetic diversity and phylogenetic relationships of different isolates^9^. While multiple genotypes of *T. gallinae* have been identified globally, their virulence and pathogenicity vary, emphasizing the need for molecular characterization of strains circulating in Egyptian pigeon populations. Comparative molecular analysis also enables the assessment of the genetic similarity between local strains and those reported globally, which may shed light on the potential virulence or epidemiological origin of infections.

This study aims to assess the prevalence, genetic diversity, and pathological effects of *T. gallinae* in domestic pigeons in Egypt. It focuses on identifying infection rates, evaluating associated risk factors, conducting molecular characterization, and examining histopathological changes in affected tissues. The findings will contribute to enhancing disease surveillance, improving control strategies, and supporting conservation efforts for pigeon populations.

## Materials and methods

### Ethical statement

This study was conducted in accordance with ethical guidelines for animal research and welfare. All procedures involving animals were reviewed and approved by the Desert Research Center Ethical Committee (Approval No. DREC-0357), in compliance with national and international standards for animal care and use, and in alignment with the ARRIVE guidelines. Sample collection was performed with minimal distress to the pigeons, ensuring compliance with ethical guidelines. Permission was obtained from farm owners and relevant authorities before conducting the study. No live animals were harmed or subjected to unnecessary suffering during the research.

### Study area

**Fig. 1:**
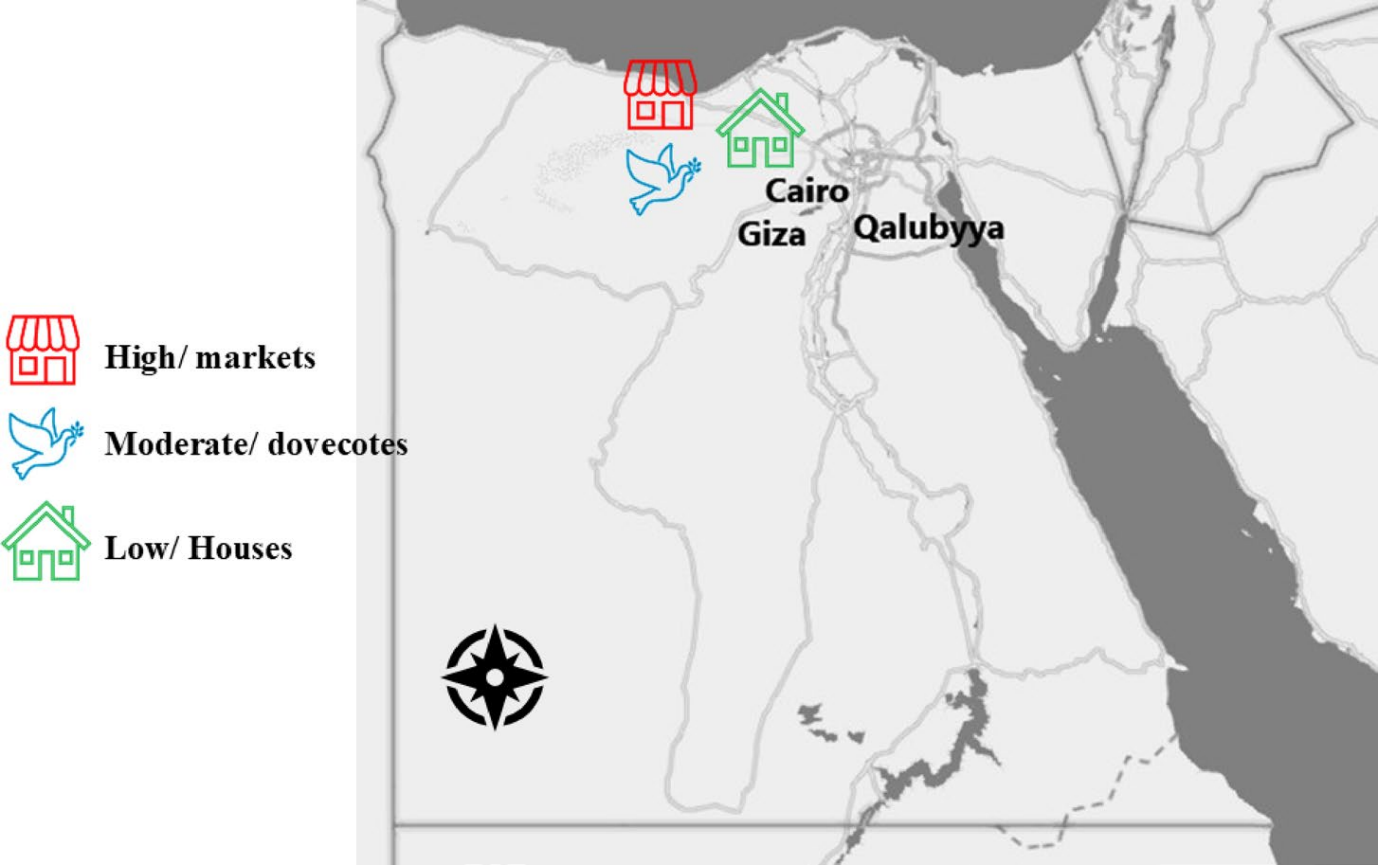
Egypt google map showed geographical distribution of *T. gallinae* infection (The map was generated using Excel, which is powered by Google Maps.). The map represents infection levels across different environments: High infection rates in markets (red), moderate infection rates in dovecotes (blue) and low infection rates in houses (green). Sample collection was conducted from live birds across these regions.

A total of 685 domestic pigeons (*Columba livia domestica*) were collected from markets, dovecotes (enclosed structures designed for breeding and sheltering pigeons), and houses in Cairo [30° 1′ 59.9988″ N, 31° 14′ 0.0024″ E], Giza [29° 58′ 35.3280″ N, 31° 7′ 52.6872″ E], and Qalubyya [30.41°N, 31.21°E] (Fig. 1) between February 2022 and November 2024. The region is characterized by a semi-arid climate (BWh under the Köppen–Geiger classification), featuring hot, dry summers and mild winters. Average summer temperatures range from 25 to 38 °C, often accompanied by low humidity and minimal precipitation. In winter months, temperatures average between 10 and 20 °C, with occasional light rain. Annual rainfall is low, typically less than 25 mm, and is concentrated during the winter months. The pigeons were classified into two age groups: squabs (< 6 months old) and adults (≥ 6 months old). The age of the pigeons was estimated based on morphological features and plumage characteristics, in consultation with experienced pigeon breeders. Additionally, the effects of seasonal variation and sex on *T. gallinae* infection rates were recorded.

### Sample collection from live bird

Oral swabs were collected by gently rolling the swab across the oral cavity and crop mucosa two to three times to maximize the retrieval of mucosal and trichomonad cells^12^. The swabs were then placed in Falcon tubes containing 0.9% saline (NaCl) for preservation and further analysis.

### Histopathological study

A histopathological study was conducted on pigeons severely infected with *T. gallinae*, confirmed through microscopic examination. A complete postmortem examination was performed, and tissue samples (0.5 cm thick) were collected from the oropharynx, proventriculus, and gizzard to assess tissue reactions to parasitic infection^13^. The specimens were fixed in 10% neutral buffered formalin for 72 h, then rinsed with tap water, dehydrated through ascending alcohol concentrations, and cleared using xylene. Five-micron thick paraffin sections were prepared, stained with hematoxylin and eosin (H&E), and examined microscopically. Tissue samples from uninfected pigeons served as control group^14,15^.

### DNA extraction

Genomic DNA (gDNA) was extracted from parasite suspensions using the QIAamp DNA Mini Kit (Cat. No. 51304, QIAGEN, USA), following the manufacturer’s protocol. The suspension was centrifuged at 800×*g* for 5 min, and the resulting pellet was washed three times with phosphate-buffered saline (PBS). The DNA extraction process involved incubating the sample with QIAGEN protease and buffer AL at 56 °C for 10 min, followed by the addition of 96% ethanol and filtration through a QIAamp Mini Spin Column. After successive washes with AW1 and AW2 buffers, DNA was eluted with buffer AE and stored at − 20 °C until further use.

### PCR amplification

Specific primers targeting the ITS1/5.8S/ITS2 region of *T. gallinae* were used, with forward primer TGCTTCAGCTCAGCGGGTCTTCC and reverse primer CGGTAGGTGAACCTGCCGTTGG^16^. Conventional PCR (cPCR) was performed using EmeraldAmp GT PCR Master Mix (Takara, Cat. No. RR310A). The reaction mixture (25 μL) consisted of 12.5 μL of master mix, 5.5 μL of PCR-grade water, 1 μL of forward primer (20 pmol), 1 μL of reverse primer (20 pmol), and 5 μL of template DNA^17^. The primers targeted the ITS1/5.8S/ITS2 region of *T. gallinae*, generating a 350 bp amplicon^12^. PCR cycling conditions included an initial denaturation at 94 °C for 5 min, followed by 35 cycles of denaturation at 94 °C for 30 s, annealing at 58.7 °C for 40 s, and extension at 72 °C for 45 s, with a final extension at 72 °C for 10 min.

### Agarose gel electrophoresis

PCR products were analyzed using 1.5% agarose gel electrophoresis in TBE buffer^18^. The gel was prepared by dissolving 1.5 g of electrophoresis-grade agarose in 100 mL of TBE buffer, heating until completely dissolved, and cooling to 70 °C before adding ethidium bromide (0.5 μg/mL). The warm agarose was poured into a gel casting tray with combs and allowed to solidify. After solidification, 20 μL of PCR amplicons, along with a Gene Ruler 100 bp DNA ladder (Fermentas, Cat. No. SM0243), were loaded into the wells. Electrophoresis was performed at 1–5 V/cm for approximately 30 min, and DNA bands were visualized using a UV transilluminator. Gel images were captured using a gel documentation system (Alpha Innotech) for further analysis.

### Phylogenetic analysis

PCR-positive samples were randomly selected for sequencing and phylogenetic analysis. Specific primers targeting the ITS1/5.8S/ITS2 region of *T. gallinae* were used, with forward primer TGCTTCAGCTCAGCGGGTCTTCC and reverse primer CGGTAGGTGAACCTGCCGTTGG^16^. The ITS1-5.8S-ITS2 rRNA gene was used as a molecular marker, and amplified PCR products were purified using a commercial purification kit (Takara Bio Inc., San Jose, California, USA) before sequencing. The obtained sequences were aligned and analyzed using bioinformatics tools such as MEGA X, ClustalW, and BLAST to assess the genetic diversity, phylogenetic relationships, and evolutionary patterns of *T. gallinae* isolates. A phylogenetic tree was constructed using the neighbor-joining or maximum likelihood method, with bootstrap analysis performed to confirm the robustness of the relationships.

### Statistical analysis

A Chi-square test of independence was performed to assess the association between *T. gallinae* infection and categorical variables, including season, age, gender, and environment (markets, dovecotes, houses). A *p*-value < 0.05 was considered statistically significant. Statistical analysis was conducted using SPSS software (Version 26, IBM, USA)^19^.

## Results

### Epidemiological results

**Fig. 2:**
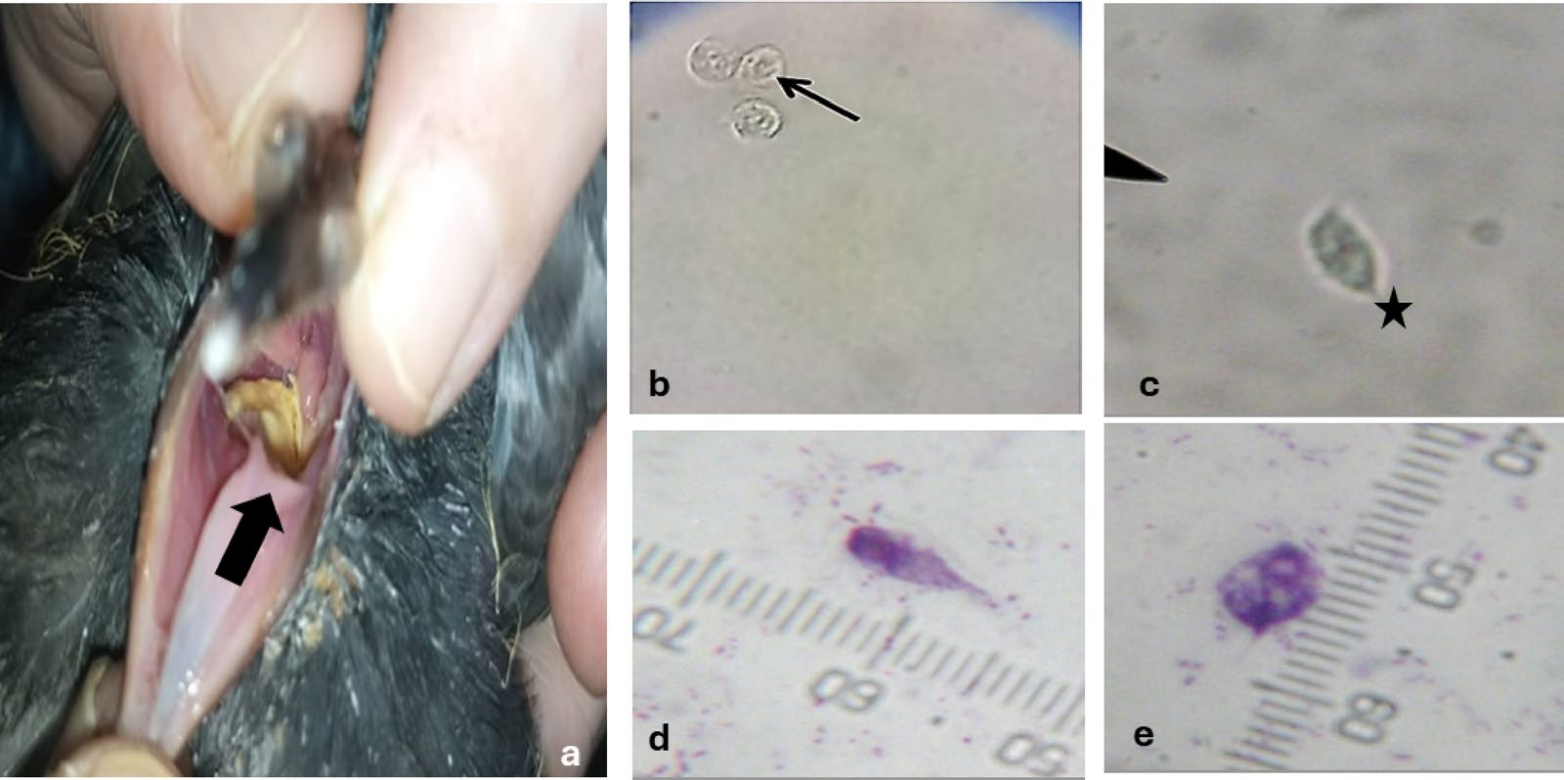
(**a**) Photograph of an infected pigeon showing a yellowish mass in the pharynx (black arrow). (**b**) Photomicrograph of a wet mount sample of *T. gallinae*, with the black arrow indicating the nucleus inside the *T. gallinae* trophozoite. (**c**) The black star highlights the anterior flagella and axostyle of T*. gallinae* (magnification ×400). (**d** & **e**) Photomicrographs showing different morphological forms of *T. gallinae* trophozoites stained with Giemsa stain (×1000), measuring 10.5–13.5 × 3–7.5 µm.

**Fig. 3:**
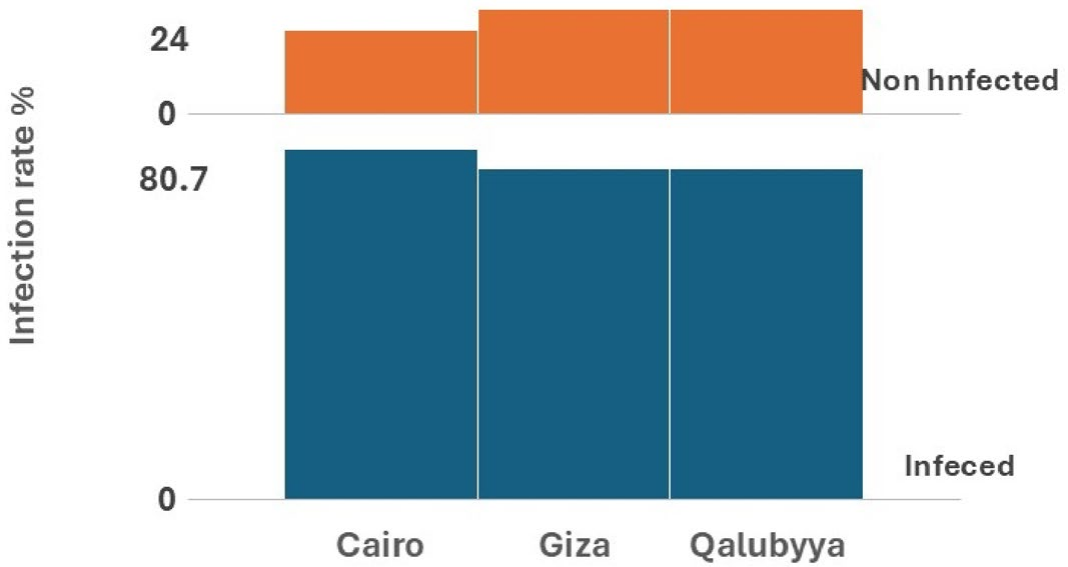
A comparative chart, generated using the multi-layer column chart feature in the KUTOOLS program for Excel, depicts the infection rates (%) among pigeons examined across various study sites.

**Fig. 4:**
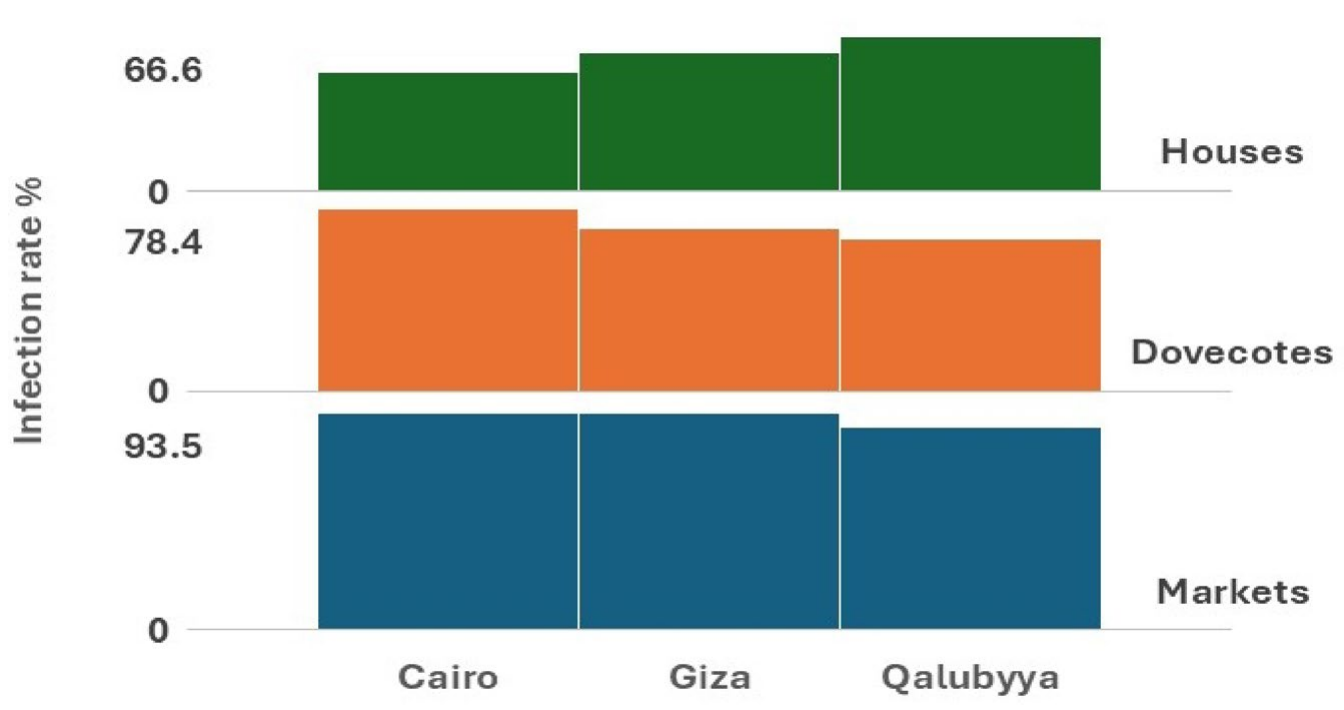
A comparison chart, created using the multi-layer column chart feature in the KUTOOLS program for Excel, illustrates the infection rates (%) of pigeons across various habitats.

Infected birds often display clinical symptoms including lethargy, progressive weight loss, and necrotic lesions affecting the upper gastrointestinal tract (Fig. 2). The results showed that the rate of infection with *T. gallinae* in *Columba livia domestica* varied across different environments and regions. A total of 685 samples were examined, with 533 found to be infected, resulting in an overall infection rate of 77.8%. Markets exhibited the highest infection rates, with Cairo reporting 114 infected out of 122 examined (93.4%), Giza with 87 infected out of 93 examined (93.5%), and Qalubyya showing 70 infected out of 80 examined (87.5%), resulting in a total market infection rate of 91.8%. Dovecotes showed moderate infection rates, with Cairo recording 73 infected out of 93 examined (78.4%), Giza 63 out of 90 (70.0%), and Qalubyya 43 out of 65 (66.1%), culminating in an overall dovecote infection rate of 72.1%. Houses demonstrated the lowest infection rates, with Qalubyya showing 20 infected out of 30 examined (66.6%), Giza 40 out of 67 (59.7%), and Cairo 23 out of 45 (51.1%), leading to a combined house infection rate of 58.4%. Regionally, Cairo had the highest total infection rate of 210 infected out of 260 examined (80.7%), followed by Giza with 190 out of 250 (76.0%) and Qalubyya with 133 out of 175 (76.0%). (Table 1 & Figs. 3 and 4).

**Table 1:** Prevalence of *T. gallinae* across different habitats.

	Exa	Markets		Exa	Dovecotes		Exa	Houses		Total examined	Total infected	%
		Inf	%		Inf	%		Inf	%			
Cairo	122	114	93.44	93	73	78.4	45	23	51.1	260	210	80.7
Giza	93	87	93.5	90	63	70	67	40	59.7	250	190	76
Qalubyya	80	70	87.5	65	43	66.1	30	20	66.6	175	133	76
Total	295	271	91.8	248	179	72.1	142	83	58.4	685	533	77.8
Chi- Square	8.25											
*p*-value	0.082											
Degree of freedom	4											
Significance	Non-Significant

The data presented in the Table 2 highlights non-significant findings regarding the distribution of cases based on season while age, and gender showed significant results across three surveyed regions: Cairo, Giza, and Qalyubia. Seasonal results indicate that summer is the most prevalent season for recorded cases, accounting for 48.2% (n = 257) of the total sample size (n = 533). In contrast, winter represented the season with the lowest number of cases, making up only 11.4% (n = 61) of the total. The remaining cases were distributed across spring (23.2%, n = 124) and autumn (17.07%, n = 91), showing intermediate levels of occurrence during these seasons.

**Table 2:** Risk factors influencing the prevalence of *T. gallinae* across surveyed sites, including season, age, and gender.

	Season								Age				Gender			
	Summer		Spring		Autumn		Winter		Squab		Adults		Female		Male	
	No	%	No	%	No	%	No	%	No	%	No	%	No	%	No	%
Cairo	103	49.04	54	25.7	33	15.7	20	9.5	166	79.4	44	20.9	160	76.1	50	23.8
Giza	84	44.2	43	22.6	38	20	25	13.1	110	57.8	80	42.1	173	91.05	17	8.9
Qalubyya	70	52.6	27	20.3	20	15.03	16	12.03	101	75.9	32	24.06	112	84.2	21	15.7
Total 533	257	48.2	124	23.2	91	17.07	61	11.4	377	70.7	156	29.3	445	83.4	88	16.6
Chi- square	4.98								23.88				16.05			
*p*-value	0.5								0.0003				0.0003			
Degree of freedom	6								2				2			
Significance	Non-significant								Significant				Significant			

Age distribution reveals that squabs (younger age group) constitute the majority of cases across all regions, representing 70.7% (n = 377) of the total sample. In contrast, adults accounted for a smaller proportion of the sample, comprising 29.3% (n = 156).

**Fig. 5:**
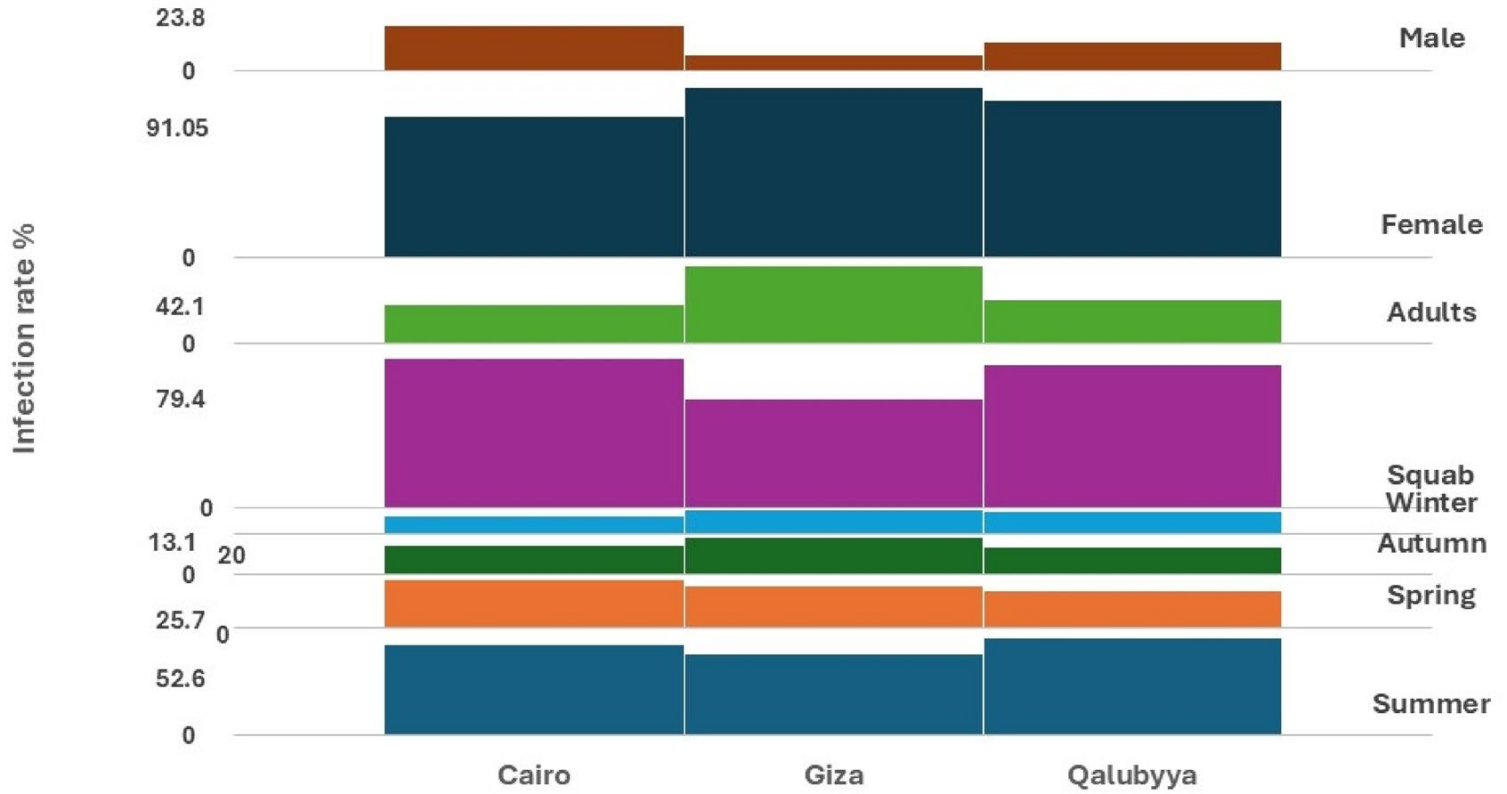
A comparison chart, created using the multi-layer column chart feature in the KUTOOLS program for Excel, illustrates the infection rates (%) during the study according to season, age and gender.

Gender distribution across the surveyed regions reveals a strong predominance of females, who accounted for 83.4% (n = 445) of the total sample. Male cases, on the other hand, represented a much smaller proportion, comprising only 16.6% (n = 88) of the total (Fig. 5).

Overall, the findings demonstrate that summer is the most dominant season for cases across the surveyed regions, with a significant majority of cases involving squabs and females.

### Histological results

**Fig. 6:**
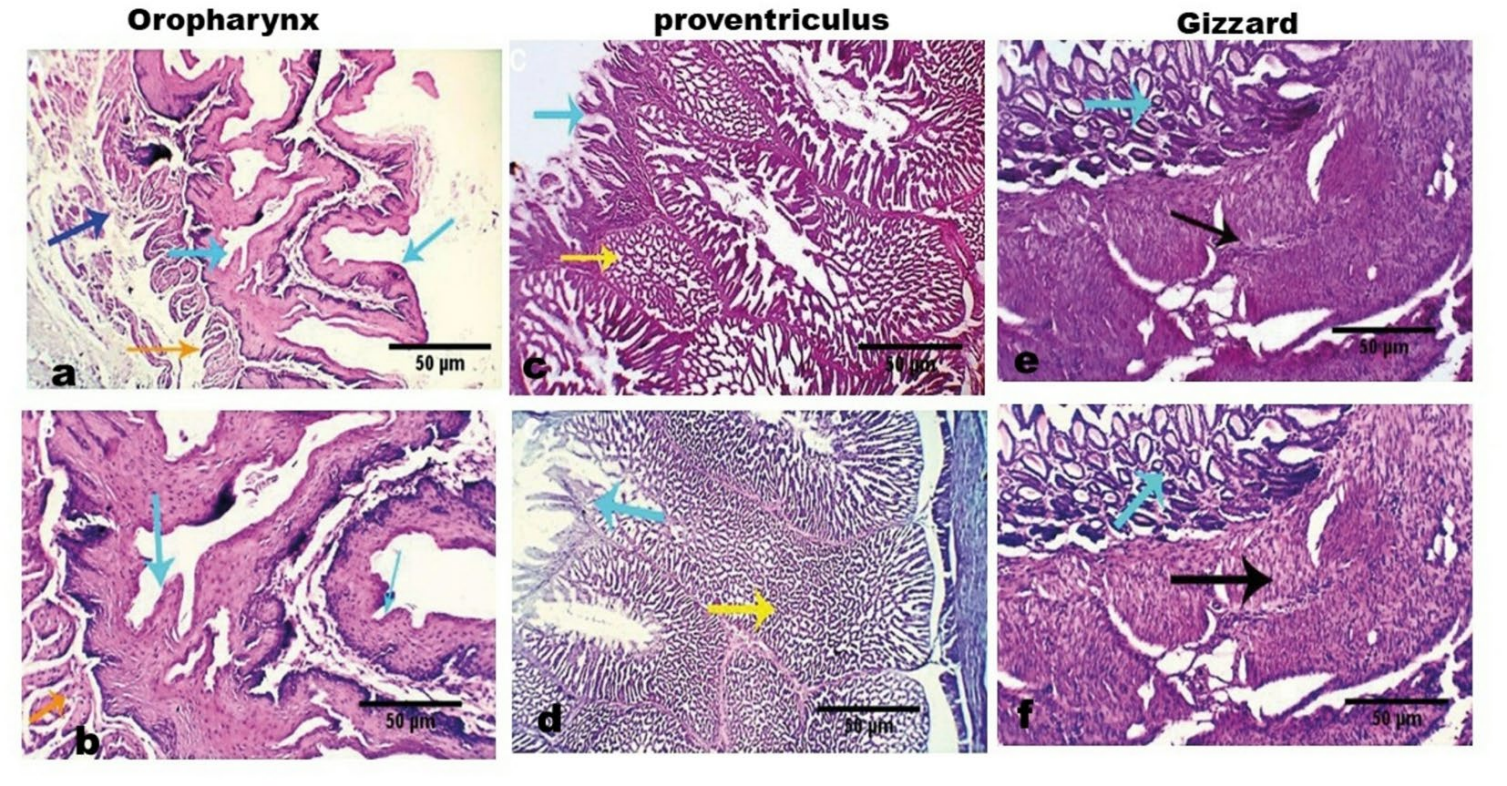
Photomicrographs of the oro-pharynx, proventriculus, and gizzard from control, *T. gallinae*-free pigeons. (**a** & **b**) Normal micro-morphological structures of the oro-pharyngeal mucosa with preserved keratinized stratified squamous epithelium (light blue arrows), underlying loose fibrous lamina propria, and submucosa (dark blue and orange arrows). (**c** & **d**) Normal proventricular mucosal lining and glands (light blue and yellow arrows). (**e** & **f**) Normal gizzard showing intact superficial glandular mucosa (light blue arrow) and muscular coats (black arrows). All examined tissues appear normal. Scale bars: 50 μm.

Examined tissue sections from healthy, uninfected pigeons, collected from the pharyngeal mucosa, proventriculus, and gizzard, revealed normal micro-morphological structures. The pharyngeal mucosa exhibited preserved keratinized stratified squamous epithelium, while the proventriculus showed an intact mucosal lining and glands. Similarly, the gizzard displayed a normal superficial glandular mucosa. The underlying loose fibrous lamina propria, submucosa, and muscular coats of all examined tissues appeared normal. No evidence of degenerative, apoptotic, necrotic, inflammatory, protozoal, or other infections was observed (Fig. 6).

Examined tissue sections from infected pigeons revealed distinct pathological changes that are highly characteristic of *T. gallinae* infection. These changes included severe destruction, necrosis, caseation, and early granulomatous reactive tissue alterations in the oro-pharyngeal mucosa. The inflammatory process extended to the underlying lamina propria, accompanied by moderate to severe vascular changes, including angio-thrombosis in some cases. The oropharyngeal lamina propria exhibited pronounced mononuclear cellular infiltration, predominantly comprising macrophages, lymphocytes, and plasma cells. Adjacent collagen fibers appeared highly degenerated, hyalinized, and focally necrotized.

**Fig. 7:**
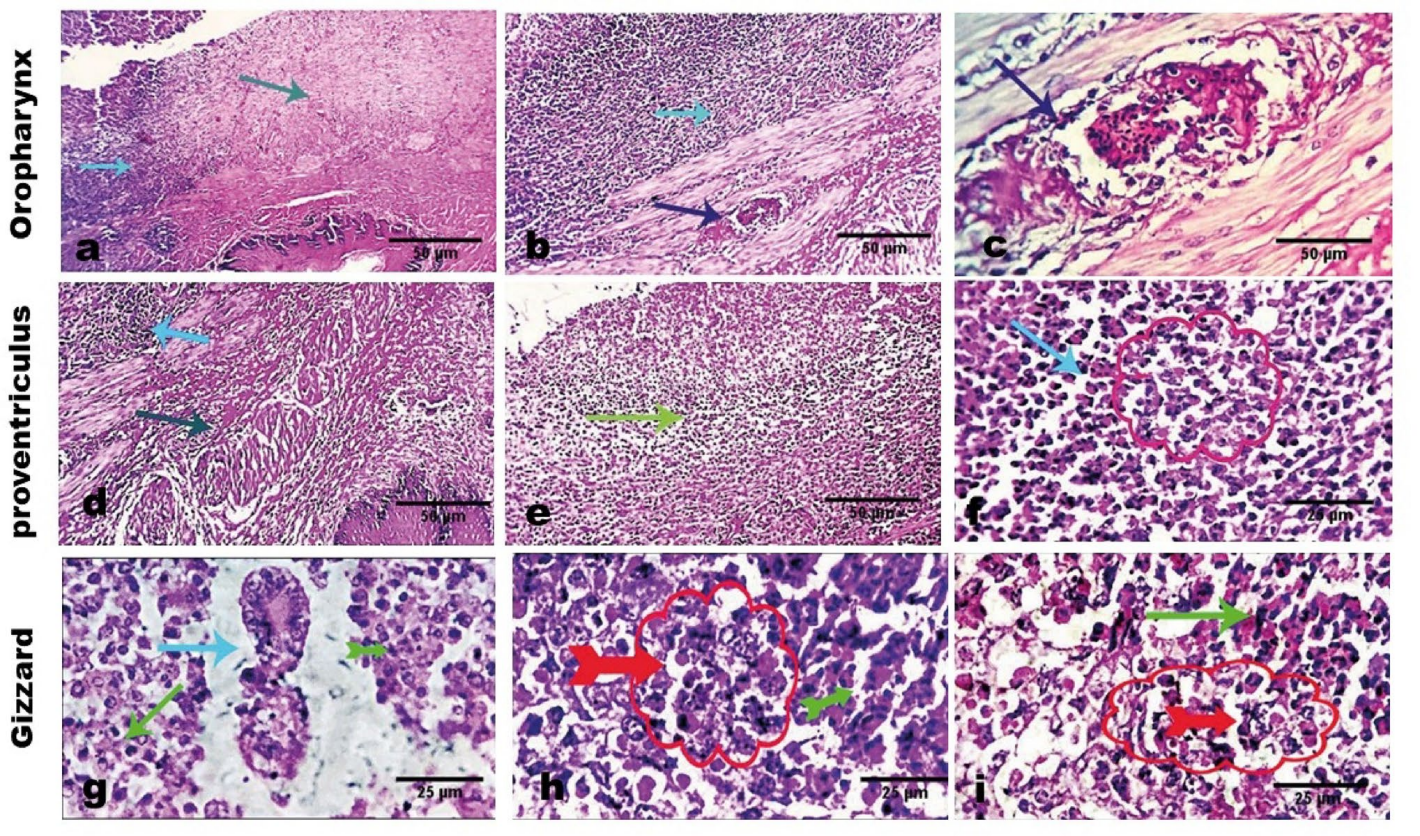
Photomicrographs of the oro-pharynx, proventriculus, and gizzard from *T. gallinae*-infected pigeons. (**a**, **b** & **c**) Marked destruction, necrosis, caseation, and early granulomatous reactive tissue changes are observed in the oro-pharyngeal mucosa (gray arrow). The inflammatory process extends to the underlying lamina propria (light blue arrows) with moderate to marked vascular changes, including angio-thrombosis in some cases (dark blue arrows). (**d**, **e** & **f**) The oropharyngeal lamina propria exhibits significant mononuclear cellular infiltration, including macrophages, lymphocytes, and plasma cells (light blue arrow). Adjacent collagen fibers appear highly degenerated, hyalinized, and focally necrotized (dark blue arrow). A mass of caseo-necrotic tissue replaces a large area of the affected mucosa (light blue arrow). (**g**, **h** & **i**) Aggregates of dead (degranulated) and living heterophils (green arrows), few lymphocytes, and occasional multinucleated giant cells (light blue arrow) are noted. Characteristic pear-shaped *T*. *gallinae* organisms are observed intermixed with inflammatory cells, particularly at the peripheral margins of the caseated material (red circles and arrows). Scale bars: 25 μm, 50 μm.

A mass of caseo-necrotic tissue replaced large areas of the affected mucosa, characterized by aggregations of dead (degranulated) and living heterophils, few lymphocytes, and occasional multinucleated giant cells. Notably, characteristic pear-shaped protozoal (*T. gallinae*) organisms were observed interspersed among the inflammatory cells, particularly at the peripheral margins of the caseated material (Fig. 7).

### Molecular results

**Fig. 8:**
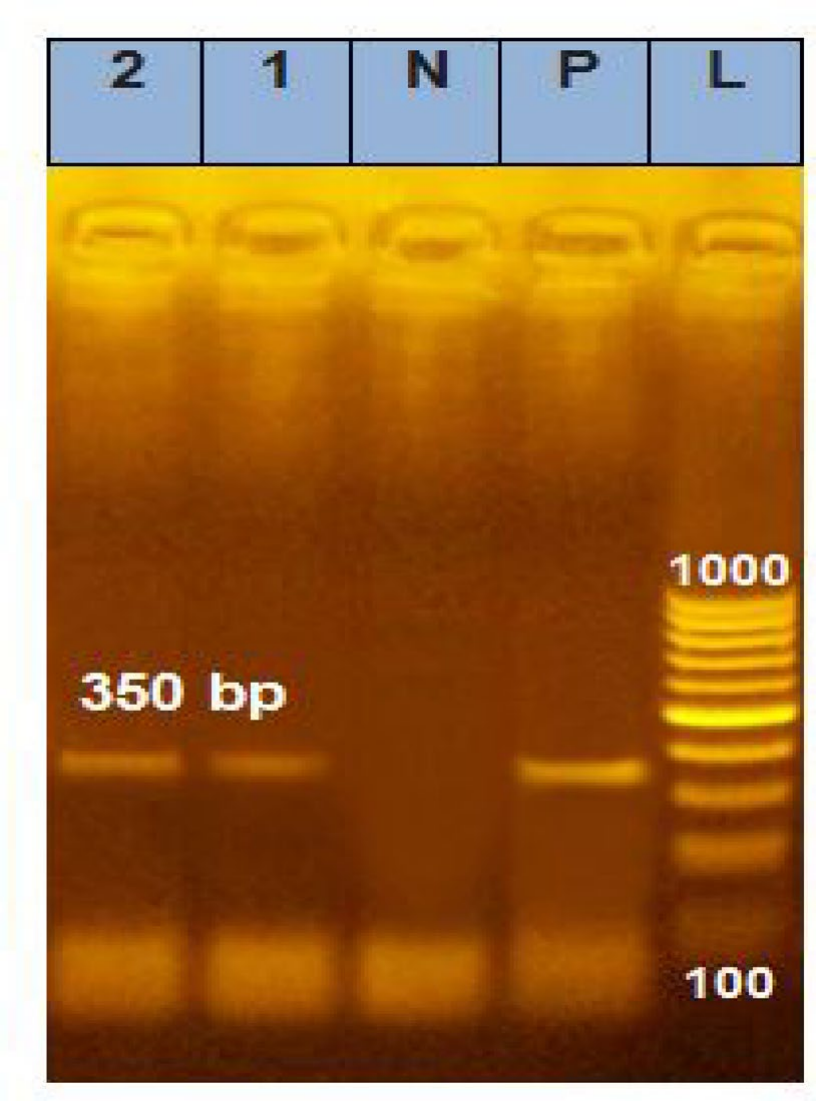
PCR-based assays targeted the ITS1-5.8S-ITS2 rRN gene for *T*. *gallinae*. L lane: 100 bp DNA ladder. Lanes 1 and 2 correspond to positive results for *T. gallinae*. Lanes P and N represent positive and negative controls, respectively.

In the present study, *T. gallinae* produced distinct bands at 350 bp in 70% of the DNA-extracted samples obtained from pigeons collected across different governorates and habitats (Fig. 8). The newly isolated nucleotide sequences from this investigation have been deposited in GenBank under the accession numbers OR498119 (*Trichomonas gallinae* strain SM1 small subunit ribosomal RNA gene, part—Nucleotide—NCBI) and OR498120 (*Trichomonas gallinae* strain KY2 small subunit ribosomal RNA gene, part—Nucleotide—NCBI).

Our data were compared using BLAST with existing sequences in GenBank, revealing 100% identity with *T. gallinae* strains from China, Germany, and Spain (Table 3).

**Table 3:** Molecular identification and comparison of *T. gallinae* strains and related species based on sequence similarity, and geographical location.

Strain	Identity %	Location	References
OR498119 *T. gallinae* SM1		Egypt	The present work
OR498120 *T. gallinae* KY2		Egypt	The present work
MH733821 *T. gallinae* BJ11	100	China	Feng et al.^16^
MH733820 *T. gallinae* BJ09	100	China	Feng et al.^16^
MH733816 *T. gallinae* RJ01	100	China	Feng et al.^16^
MH733819 *T. gallinae* BJ07	100	China	Feng et al.^16^
KJ721784 *T. gallinae* Tai1	100	China	Jiang et al.^20^
U86614 *T. gallinae*	100	–	Felleisen^21^
EU881912 *T. gallinae* P4406	100	Germany	Wiemann et al.^22^
KX459475 *T. gallinae* RTNS7	100	Spain	Marx et al.^23^
KX459474 *T. gallinae* RTNS6	100	Spain	Marx et al.^23^
KX459497 *T. gallinae* TT-ES_34	100	Spain	Marx et al.^23^
KX459497 *T. gallinae* WP4	100	Spain	Marx et al.^23^
EU8881911 *T. gallinae* P1807	98.9	Germany	Wiemann et al.^22^
KJ721785 *T. gallinae* Tai 2	98.9	China	Jiang et al.^20^
MH733817 *T. gallinae* BJ02	98.9	China	Feng et al.^16^

High sequence similarity percentages (above 90%) were observed with other known strains, such as *T*. *stableri* (93.3%), *T*. *vaginalis* (92.5%), and *T. brixi* (94.4%), confirming their conserved nature across locations. The zoonotic potential of *Trichomonas* species is evident from the identification of *T. tenax* (94.4%) and *T. canistomae* (74.7%). Furthermore, non-Trichomonas species were also identified, including *Histomonas meleagridis* (71.7%), *Trigonella sp*. (74.2%), and *Tetratrichomonas prowazekii* (71.4%) collected worldwide. A lower sequence similarity was observed for *Pentatrichomonas hominis* (69.7%) (Table 4).

**Table 4:** Molecular identification and comparison of *Trichomonas* spp. strains and related species based on sequence similarity, and geographical location.

Strain	Identity %	Location	Ref
KC215389 *T. stableri*	93.3	California	Girard et al.^24^
KP987799 *T. vaginalis* ADUTV03	92.5	Turkey	Ertabaklar et al.^25^
KX977527 *T. brixi* OCa43	94.4	Czech Republic	Kellerová and Tachezy^26^
KX186997 *T. tenax* H95	94.4	Brazil	Cembraneli et al.^27^
AJ784786 *T. canistomae*	74.7	Poand	Turkowicz^28^
KJ863548 *H. meleagridis* YZ9	71.7	China	Qu^29^
JX132891 *Trigonella* CEas226	74.2	France	Gouba et al.^30^
OK584282 *Tetratrichomonas prowazeki* JL	71.4	North America, Asia, and Africa	Céza et al.^31^
MN173976 *P. hominis* CCH_ITS3	69.7	China	Zhang et al.^32^

**Fig. 9:**
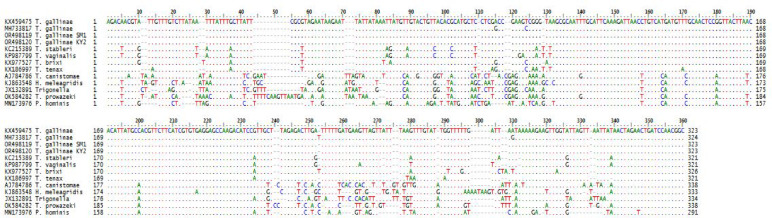
Multiple sequence alignment showing conserved regions, nucleotide substitutions, and gaps among isolated in the present study and related sequences. Dots indicate base identical to those in obtained isolates.

**Fig. 10:**
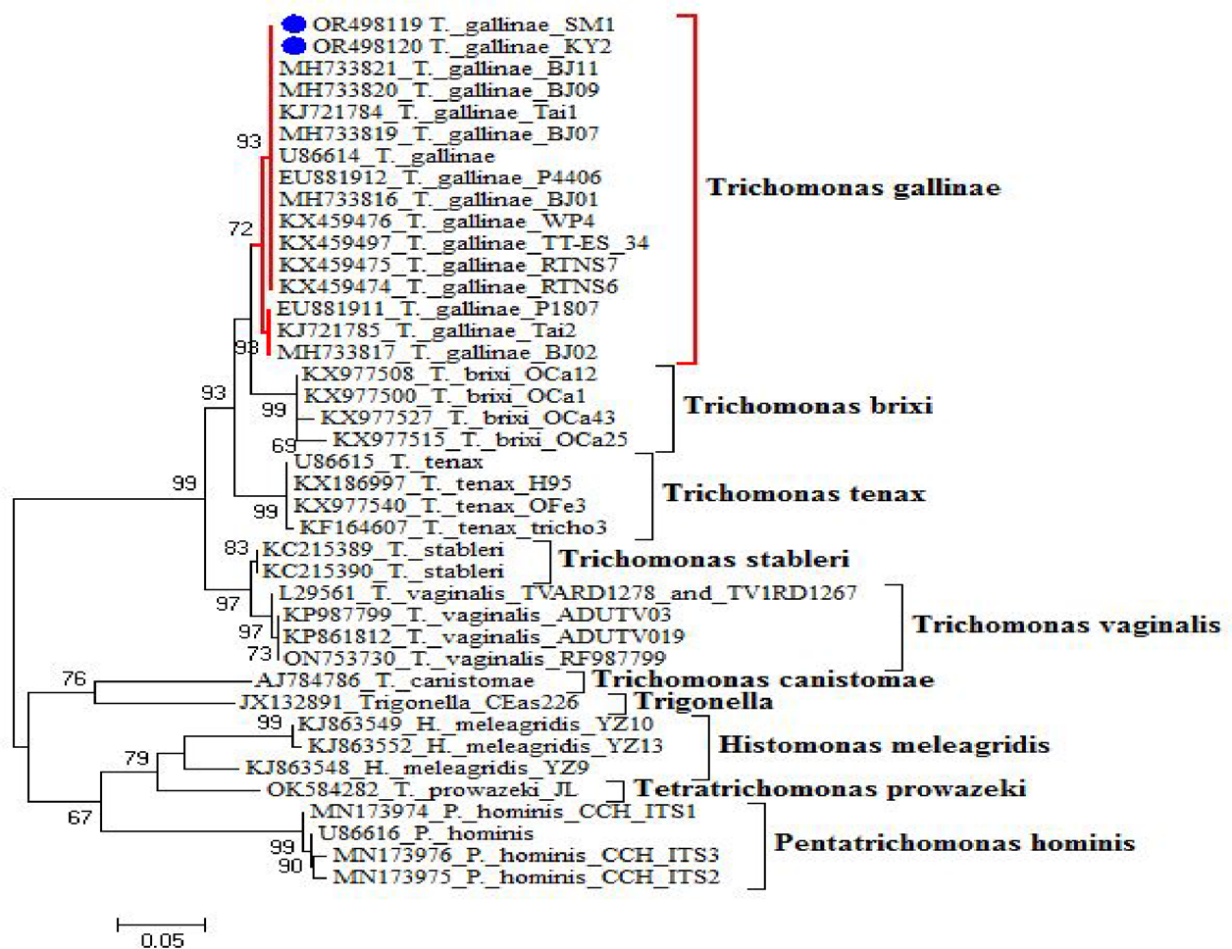
Phylogenetic tree showing evolutionary relationships among *T. gallinae* strains, including the newly recorded *T. gallinae* SM1 and *T. gallinae* KY2 (blue dots), and related species. Bootstrap values indicate branch support. Scale bar: 0.05 substitutions per site.

The multiple sequence alignment (MSA) revealed that the two newly recorded strains (T*. gallinae* SM1 and T*. gallinae* KY2) show high similarity to the reference strain *T. gallinae* (KX459475), with minor nucleotide differences. Substitutions were identified at positions 50 (A → T in KY2), 110 (T → C in SM1), and 200 (G → A in both SM1 and KY2), along with small gaps around position 150. Other sequences, such as *T. stableri* (KC135399) and *T. tenax* (KU877697), displayed more pronounced differences, including substitutions at positions 70 (C → A), 180 (T → G), and 220 (A → T), and longer gaps between positions 140–160. Non-*T. gallinae* species (e.g., *T. vaginalis* and *T. bryoni*) exhibited higher levels of sequence divergence, with multiple mismatches and insertion/deletion events throughout the alignment, as shown in Fig. 9. The phylogenetic tree based on the ITS1/5.8S/ITS2 rRNA sequences showed that the *T. gallinae* sequences clustered together into a single well-supported clade, as shown in Fig. 10.

## Discussion

In our study, the overall incidence of *T. gallinae* infection in examined pigeons was 77.8% (533/685) across the surveyed regions and environments, indicating a high prevalence. This finding aligns closely with the 73% infection rate reported by Tuska-Szalay et al.^33^ in four columbiform bird species in Hungary. However, our results show a higher prevalence compared to previous studies, other studies revealed lower infection rates, such as Abd El-Rahman et al.^34^, Afrin et al.^35^, Mohamed et al.^36^ and Salem et al.^37^ reported infection rates of 68.92%, 57.4%, 64%, and 25% respectively, in pigeons from different regions, including Qalubyya Governorate (Egypt), Jessore District (Bangladesh), and Giza Governorate (Egypt). Similarly, Fadhil and Faraj^38^ detected a 58% infection rate in Iraq using both microscopy and PCR techniques. Conversely, lower prevalence rates have been recorded in other studies. Alrefaei^39^ reported an infection rate of 26.58% in falcons in Saudi Arabia, while Elbahy et al.^8^ observed only 4.3% in domestic pigeons from Sadat, Egypt. The lowest prevalence was documented by El-Khatam et al.^40^, who found just 1.9% in pigeons from Minoufiya Governorate, Egypt. These variations in infection rates may be attributed to multiple factors, including geographic location, host species, diagnostic methodologies, and environmental conditions affecting parasite transmission^40^. Differences in sample sizes, bird populations, and health status, along with climatic conditions, seasonal variation, host resistance, dietary habits, bird age, and housing conditions, also contribute to the observed discrepancies^41^.

During the study, the highest *T. gallinae* infection rates were observed in markets, where birds from diverse sources are densely packed, increasing direct contact and exposure to contaminated food and water. Overcrowding, poor sanitation, and stress further facilitated transmission. In dovecotes, infection rates were lower than in markets due to prolonged close contact, shared nesting sites, and parent-to-offspring transmission, though controlled housing conditions may help limit the spread. In houses, infection rates were comparatively lower, as birds were kept in smaller numbers with less exposure to external sources. Overall, the prevalence was significantly higher in pigeons kept under poor conditions compared to those in good or excellent conditions, as reported by Jiang et al.^20^.

Our results indicate that seasonal variations may influence *T. gallinae* infection rates, with the highest prevalence recorded during the warmer months of spring and summer, whereas the lowest incidence was observed in autumn and winter. These findings align with those of Fadhil and Faraj^38^, Elbahy et al.^8^, and Salem et al.^42^ who also reported a higher infection rate in spring compared to winter. This seasonal pattern may be attributed to the warm breeding period of pigeons, which leads to an increase in newly hatched squabs. These young pigeons are particularly vulnerable to infection through maternal transmission via contaminated crop milk^43^.

In the present study, the prevalence of *T. gallinae* was higher in squabs (young pigeons) than in adults, aligning with the findings of Al-Sadi and Hamodi^44^, who reported a higher infection rate in younger pigeons in Mosul, Iraq. Similarly, Feng et al.^16^ observed the highest prevalence in nestling pigeons in China, followed by adolescent pigeons and breeding pigeons. However, our results contrast with those of Bunbury et al.^45^, who found that pigeons aged 3–12 months had a lower infection rate compared to those over one year old. Additionally, Fadhil and Faraj^38^ reported a higher prevalence in adult pigeons than in younger ones in Baghdad, Iraq. The aggregation of pigeons around feeders and water sources, coupled with overcrowding stress, may contribute to increased infection rates^46^.

A significantly higher prevalence of *T. gallinae* infection was detected in female pigeons compared to males, aligning with previous studies. Begum et al.^12^, Abd El-Rahman et al.^34^, and Saikia et al.^47^ similarly reported higher infection rates in females, with males exhibiting the lowest incidence. The reason for this higher prevalence in females remains unclear, but it may be associated with sex hormones that increase susceptibility to infection^47^. Conversely, Al-Sadi and Hamodi^44^ reported a greater occurrence of trichomoniasis in male pigeons than in females among free-living urban pigeons. Similarly, Al-Barwari and Saeed^48^ found a slightly higher infection rate in males than in females in Iraq. However, Bunbury et al.^45^ and Fadhil and Faraj^38^ observed no significant difference in infection rates between sexes, suggesting that both males and females have an equal likelihood of contracting *T. gallinae*.

Histopathological examination of the oro-pharynx, proventriculus, and gizzard in *T. gallinae*-infected pigeons revealed severe tissue destruction, necrosis, caseation, and early granulomatous reactions in the oro-pharyngeal mucosa. The inflammatory response extended to the lamina propria, accompanied by moderate to severe vascular changes, including angiothrombosis in some cases. These findings are consistent with those of Begum et al.^12^, who observed yellowish to grayish necrotic lesions in the proventriculus. Similarly, Abd El-Rahman et al.^34^ in Egypt reported necrosis and inflammatory cell infiltration, predominantly eosinophils, in the esophageal mucosa, aligning with our study. Additionally, our findings revealed intense mononuclear cell infiltration in the oropharyngeal lamina propria, comprising macrophages, lymphocytes, and plasma cells. Adjacent collagen fibers exhibited marked degeneration, hyalinization, and focal necrosis. These results correlate with the observations of Widiawati et al.^49^, who reported inflammatory cell infiltration in the submucosal layer of the proventriculus. Furthermore, they are in agreement with Fadhil and Faraj^38^, who described multiple caseous necrotic lesions primarily affecting the larynx and esophagus, along with diffuse mononuclear cell infiltration and congestion in the lamina propria. The severity of lesions caused by *T. gallinae* in the upper digestive tract can vary from mild mucosal inflammation to severe submucosal involvement^50^. The extensive caseo-necrotic tissue replacing large areas of the affected mucosa in this study may result from a traumatic reaction to *T. gallinae* infection^51^. The pathogenicity of *T. gallinae* is also influenced by multiple factors, including previous pathogen exposure (protective immunity) and the overall immunocompetence of the host population^51^. Additional contributing factors include age, co-infections, genetic variability, habitat conditions, regional differences, transmission routes, and food availability^2^. Although the current study focused on the upper digestive tract—recognized as the primary site of lesion development in *Trichomonas gallinae* infections in pigeons—future investigations may benefit from extending histopathological assessments to other organs such as the liver and kidney, particularly to explore potential systemic effects in highly virulent cases.

The present study utilized PCR-based molecular assays targeting the ITS1/5.8S/ITS2 rRNA gene to identify and characterize *T. gallinae* strains isolated from pigeons across different Egyptian governorates and habitats. The PCR analysis produced distinct 350 bp bands in 70% of the DNA-extracted samples, confirming the presence of *T. gallinae*. Subsequent sequencing and BLAST analysis of the amplified ITS1/5.8S/ITS2 rRNA gene identified two distinct *T. gallinae* strains, designated as OR498119 SM1 and OR498120 KY2, which were deposited in GenBank. These sequences exhibited 100% identity with previously reported *T. gallinae* strains from China^16,20^ Germany^22^ and Spain^23^ indicating the highly conserved nature of the ITS1/5.8S/ITS2 rRNA gene in this species. The presence of *T. gallinae* strains in Egypt, which exhibited high genetic similarity to isolates from Spain, Germany, and China, suggests a possible link to avian migration routes. European Columbidae species, particularly turtle doves (*Streptopelia turtur*), migrate across Spain, Germany, and other parts of Europe, passing through the Mediterranean region, North Africa, and the Middle East on their way to their wintering grounds in Africa. Additionally, pigeons and doves from China may contribute to the spread of *T. gallinae* through Asian migratory flyways, traveling southward through Central Asia and into regions of the Middle East^25^. Beyond *T. gallinae*, the molecular analysis detected high sequence similarities (above 90%) with other *Trichomonas* species, further supporting the conserved nature of the ITS1/5.8S/ITS2 rRNA gene across different taxa. Notably, *T. stableri* (93.3%)^24^, *T. vaginalis* (92.5%)^25^, *T. brixi* (94.4%)^26^ exhibited considerable sequence homology, emphasizing the evolutionary relatedness among trichomonads. The zoonotic potential of some species was evident, as *T. tenax* (94.4%)^27^, and *T. canistomae* (74.7%)^28^. The observed identity may be attributed to the notable genetic homogeneity within the genus *Trichomonas*^21,52^. Notably, these findings indicate that the ITS1-5.8S rRNA-ITS2 region is a reliable marker for inter-species differentiation.

Our findings confirm the widespread prevalence, genetic conservation, and significant pathogenic effects of *T. gallinae*, emphasizing the need for enhanced surveillance, control measures, and further research on its transmission dynamics and potential impact on avian health.

## Conclusion

The study underscores the widespread occurrence of *T. gallinae* in urban and commercial pigeon populations, with environmental, seasonal, and biological factors influencing infection dynamics. The detection of novel genetic variants and severe histopathological damage emphasizes the need for enhanced disease surveillance and biosecurity measures, particularly in high-risk environments such as markets. Given the potential role of migratory birds in the transmission and genetic exchange of *T. gallinae*, further research is required to explore the epidemiological link between domestic, wild, and migratory avian hosts. Future studies should also focus on advanced molecular markers for improved strain differentiation, as well as investigating potential zoonotic implications. Strengthening control strategies, health monitoring programs, and awareness efforts will be crucial in mitigating the impact of *T. gallinae* infections on pigeon populations and beyond.

## Electronic supplementary material

Below is the link to the electronic supplementary material.

Supplementary Material 1

## Data Availability

All data supporting the findings of this study are available within the paper.
