# Supplementary figures and images for: Prevalence, molecular characterization, and histopathological impact of Trichomonas gallinae in domestic pigeons from Northeastern Egypt
Source: Sci Rep. 2025 Aug 4;15:28333. doi: 10.1038/s41598-025-12854-2 (PMC12322151; doi:10.1038/s41598-025-12854-2)

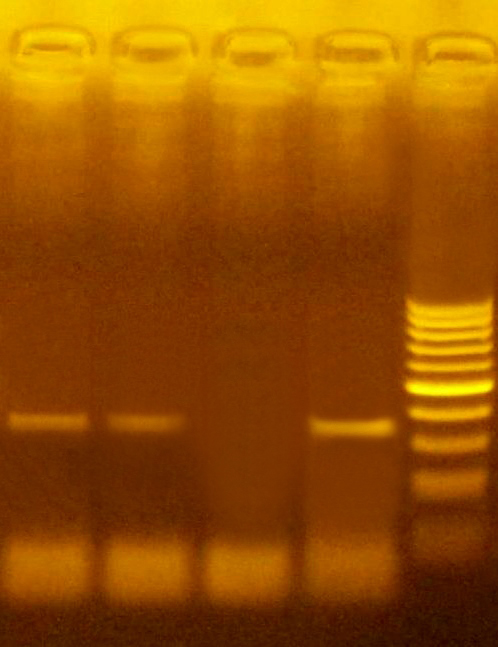


PCR-based assays targeted the ITS1-5.8S-ITS2 rRN gene for *T*. *gallinae*.

Supplement: Supplementary file 1 — Supplementary Material 1 [file 41598_2025_12854_MOESM1_ESM.docx]
